# Posterior Reversible Encephalopathy Syndrome: The Eyes Reflecting Systemic Health

**DOI:** 10.7759/cureus.82754

**Published:** 2025-04-21

**Authors:** Vinita Ramnani, Sakshi Ramnani, Divya Trivedi, Prateek Deo, Rahul Jain

**Affiliations:** 1 Ophthalmology, Bansal Hospital, Bhopal, IND; 2 Ophthalmology, Sankara Nethralaya, Chennai, IND; 3 Ophthalmology, Sadguru Sankalp Netra Chikitsalaya, Lateri, IND; 4 Rheumatology, Bansal Hospital, Bhopal, IND; 5 Radiology, Bansal Hospital, Bhopal, IND

**Keywords:** hypertension, immunosuppressant, neuroimaging, posterior reversible encephalopathy syndrome, retinopathy

## Abstract

Posterior reversible encephalopathy syndrome (PRES) is a condition marked by reversible subcortical brain swelling, often accompanied by acute neurological symptoms. It is commonly associated with renal failure, blood pressure fluctuations, cytotoxic drugs, autoimmune disorders, and preeclampsia or eclampsia. We report the case of a 34-year-old female who developed sudden blurring of vision in both eyes after an episode of accelerated hypertension. Neurological imaging revealed features of PRES, and fundoscopy showed hypertensive retinopathy in both eyes. She was managed aggressively in the ICU, and her best corrected visual acuity improved after one week, with gradual resolution of the hypertensive retinopathy. PRES typically follows a benign course, with visual deficits often resolving as the underlying systemic condition improves.

## Introduction

Posterior reversible encephalopathy syndrome (PRES) is characterized by blurred vision, headache, nausea, vomiting, and/or other neurological symptoms, along with vasogenic edema in the parieto-occipital regions of the brain [1]. The most common triggers for PRES include hypertension, renal failure, preeclampsia, eclampsia, autoimmune conditions, and cytotoxic agents, with endothelial dysfunction implicated in the pathogenesis of the syndrome [1]. Ophthalmological deficits are observed in 35-40% of patients with PRES, ranging from decreased visual acuity and visual field deficits to color vision abnormalities, visual hallucinations, and cortical blindness [2]. We report a case of a 34-year-old female with PRES who developed hypertensive retinopathy in both eyes (grade three in the right eye and grade four in the left eye), which played a key role in diagnosing the condition.

## Case presentation

**Figure 1:**
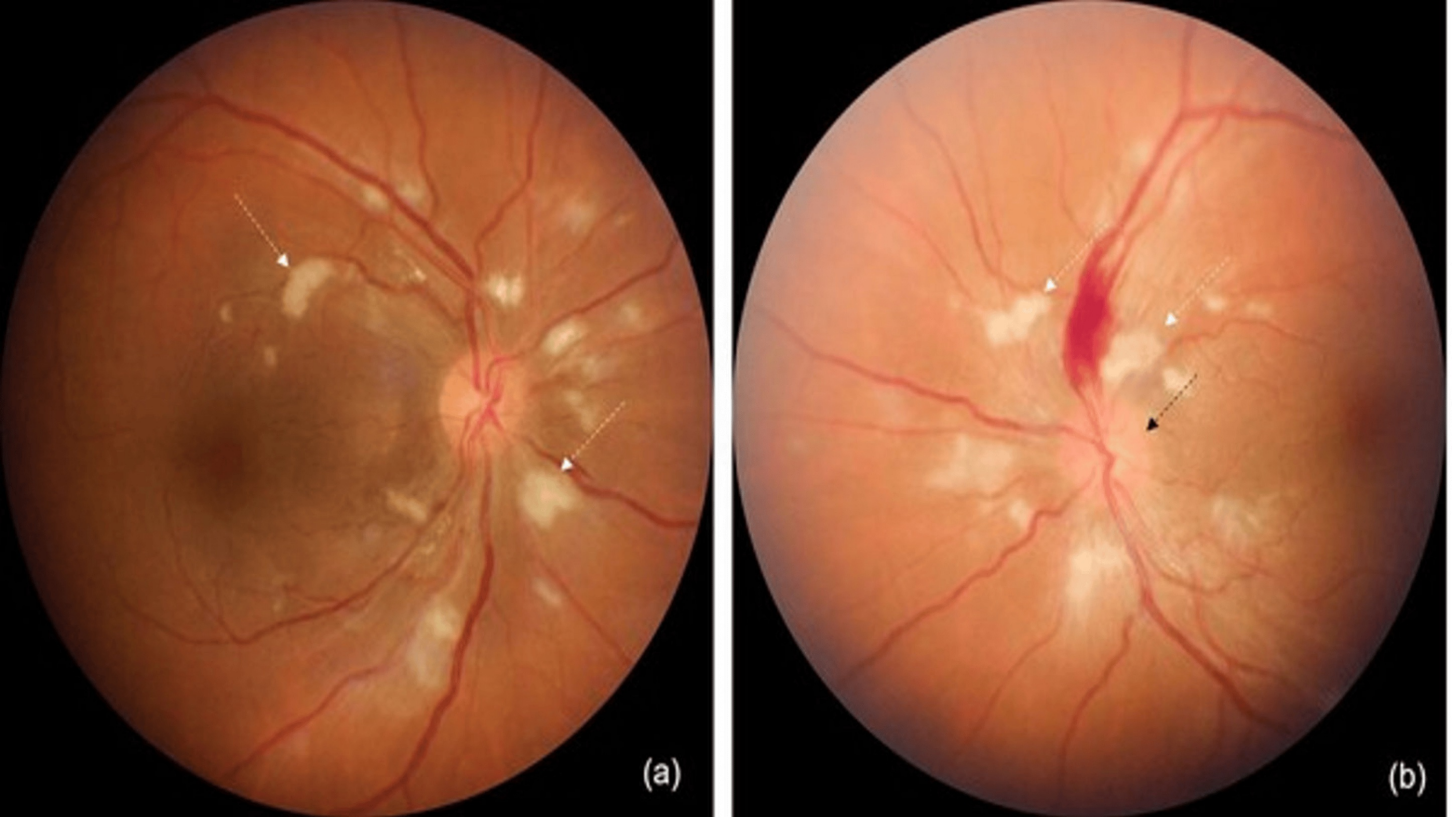
Fundus photographs showing hypertensive retinopathy (a, b) Cotton wool spots (white arrows) are present in both eyes. (b) Frank disc edema (black arrow) and a flame-shaped hemorrhage are observed in the left eye.

A 34-year-old female experienced sudden, painless vision loss in both eyes, accompanied by severe headache and vomiting. She had recently been diagnosed with dermatomyositis and had received one gram of rituximab intravenously the day before. On examination, she was found to have accelerated hypertension, with a blood pressure of 240/120 mm Hg. She was immediately given an intravenous labetalol injection and nitroglycerin infusion, then admitted to the ICU for further management. On bedside evaluation, her unaided visual acuity was counting fingers at three meters in both eyes, and the anterior segment examination was unremarkable. Fundus examination revealed cotton-wool spots in both eyes, a large flame-shaped hemorrhage superior to the optic disc in the left eye, and disc edema (Figure 1a, 1b). These findings were suggestive of hypertensive retinopathy in both eyes - grade three in the right eye and grade four in the left eye.

**Figure 2:**
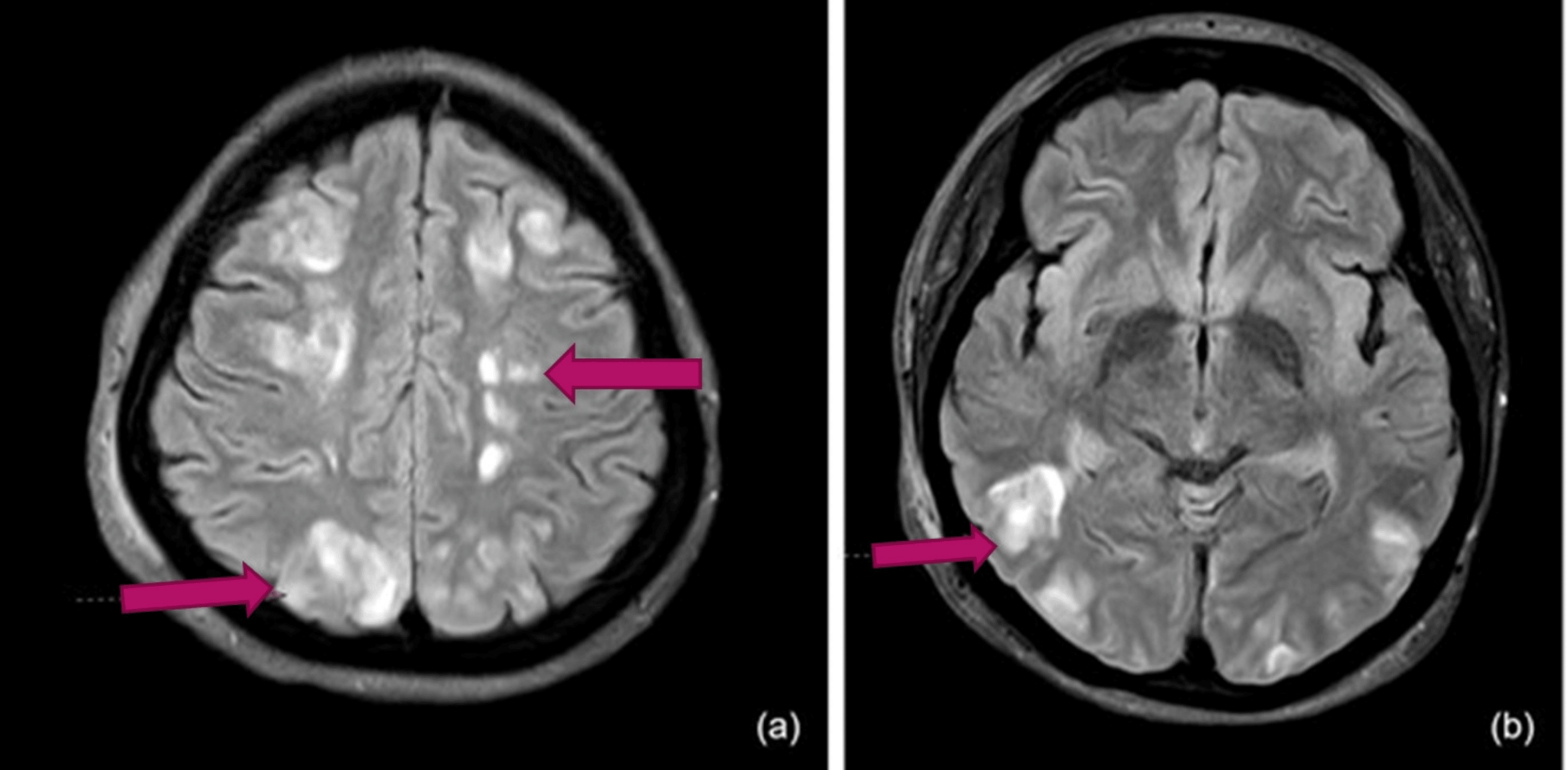
MRI of the brain Multiple near-symmetrical small areas of T2/FLAIR hyperintensity scattered across the bilateral cerebral hemispheres, including the cortex, subcortical, and deep white matter (red arrows). FLAIR, fluid-attenuated inversion recovery

The next day, she developed seizures, which were treated with intravenous levetiracetam. Her MRI revealed multiple near-symmetrical small areas of altered T2/fluid-attenuated inversion recovery (FLAIR) hyperintensity, scattered in the cortex, subcortical, and deep white matter of both cerebral hemispheres (Figure 2a, 2b). Similar foci were observed in both the basal ganglia, the splenium of the corpus callosum, and the bilateral cerebellar white matter. These features were likely secondary to vascular injury and suggested the diagnosis of PRES. The following day, her creatinine levels were found to be deranged, and she developed secondary anuria, requiring hemodialysis. After a week of intensive care, the frequency of hemodialysis gradually decreased. Her unaided visual acuity improved to 6/6, N6 in both eyes, and fundoscopy revealed complete resolution of disc edema and hypertensive retinopathy. She was safely discharged from the hospital with no visual or neurological deficits. 

On her last follow-up, a renal biopsy was performed. The specimen revealed patchy cortical necrosis, severe acute tubular injury, widespread ischemic glomerular changes, and thrombotic microangiopathy, all suggestive of end-stage chronic kidney disease. She is currently scheduled for a renal transplant.

## Discussion

PRES is a clinico-radiological diagnosis characterized by acute to subacute neurological symptoms, along with distinctive findings on neuroimaging. It has been observed in cases with severe blood pressure fluctuations and abrupt arterial hypertension, as well as in patients with eclampsia, autoimmune disorders, and those on immunosuppressant medications [3]. PRES is reported to occur more frequently in women and can present at any age. Clinical features typically include headache, visual deficits, altered levels of consciousness, and, in extreme cases, seizures [3]. Neuroimaging often reveals near-symmetrical vasogenic edema involving the subcortical white matter and cortex, most clearly seen on MRI with FLAIR sequences. Our case demonstrated these typical findings, as evidenced by the MRI images (Figure 2a, 2b).

The pathophysiology of PRES has been linked to endothelial injury, which may occur due to abrupt changes in blood pressure or direct toxic effects of cytokines. Once the endothelium is compromised, the blood-brain barrier breaks down, leading to vasogenic brain edema [4].

The relationship between PRES and malignant hypertension is often bidirectional. Severe hypertension can result in endothelial dysfunction and the breakdown of cerebral autoregulation, triggering PRES. Conversely, PRES may exacerbate neurogenic hypertension in some cases. In our patient, the presence of grade 4 hypertensive retinopathy and severely elevated blood pressure suggests that malignant hypertension was the precipitating factor, with PRES developing as a neurotoxic manifestation of the crisis [4,5].

In the setting of malignant hypertension, abrupt surges in blood pressure overwhelm cerebral autoregulation, particularly in the posterior circulation, leading to vasogenic edema, which is the hallmark of PRES. While hypertensive encephalopathy is primarily a clinical diagnosis, PRES adds a radiologically defined endpoint to the continuum. This reflects the current understanding that PRES may occur within the pathophysiological continuum of hypertensive emergencies [4,6].

There is no specific treatment for PRES, but the disorder is typically reversible when the underlying cause is eliminated or treated [4].

Clinically and radiologically, PRES often overlaps with reversible cerebral vasoconstriction syndrome (RCVS). PRES occurs in 9%-38% of RCVS cases, and 87% of PRES cases exhibit angiographic features consistent with RCVS, such as diffuse vasoconstriction, vessel pruning, and focal vasculopathy [7]. The main difference lies in their triggers - PRES is associated with uncontrolled hypertension, renal failure, exposure to cytotoxic agents, or preeclampsia, whereas RCVS is linked to immunosuppression, the puerperium, and exposure to vasoactive amines [8].

With prompt diagnosis and treatment, most cases of PRES resolve within a week [9]. However, neurological sequelae remain in 10-20% of patients [10], and mortality has been observed in 3-6% of patients [11]. This may be due to intracranial hemorrhage, increased global intracranial pressure, diffuse cerebral edema, posterior fossa edema with brainstem compression, or acute hydrocephalus [12].

## Conclusions

PRES typically follows a benign course, with most patients experiencing a complete resolution of symptoms. Patients with accelerated hypertension and those undergoing immunosuppressant therapy should be closely monitored and considered for neuroimaging. Ophthalmologists should remain aware of the potential associations between PRES and visual deficits, with hypertensive retinopathy being one of the possible causes of impaired vision.
